# Suppressing Multi-Channel Ultra-Low-Field MRI Measurement Noise Using Data Consistency and Image Sparsity

**DOI:** 10.1371/journal.pone.0061652

**Published:** 2013-04-23

**Authors:** Fa-Hsuan Lin, Panu T. Vesanen, Yi-Cheng Hsu, Jaakko O. Nieminen, Koos C. J. Zevenhoven, Juhani Dabek, Lauri T. Parkkonen, Juha Simola, Antti I. Ahonen, Risto J. Ilmoniemi

**Affiliations:** 1 Institute of Biomedical Engineering, National Taiwan University, Taipei, Taiwan; 2 Department of Biomedical Engineering and Computational Science, Aalto University School of Science, Espoo, Finland; 3 Department of Mathematics, National Taiwan University, Taipei, Taiwan; 4 Elekta Oy, Helsinki, Finland; University of Maryland - College Park, United States of America

## Abstract

Ultra-low-field (ULF) MRI (*B*_0_ = 10–100 µT) typically suffers from a low signal-to-noise ratio (SNR). While SNR can be improved by pre-polarization and signal detection using highly sensitive superconducting quantum interference device (SQUID) sensors, we propose to use the inter-dependency of the *k*-space data from highly parallel detection with up to tens of sensors readily available in the ULF MRI in order to suppress the noise. Furthermore, the prior information that an image can be sparsely represented can be integrated with this data consistency constraint to further improve the SNR. Simulations and experimental data using 47 SQUID sensors demonstrate the effectiveness of this data consistency constraint and sparsity prior in ULF-MRI reconstruction.

## Introduction

MRI has become an indispensible resource in clinical medicine because of its non-invasiveness and excellent contrast between soft tissues without using ionizing radiation. From the clinical perspective, MRI still faces significant challenges. First, a strong magnet is usually required to generate a sufficient magnetization to be detected by NMR techniques. The price of a high-field magnet (1.5 T and above) constitutes a major part of the cost of an MRI system. Its weight also excludes MRI applications in mobile or remote settings, such as ambulance, space station, or battlefield. Obese patients cannot always obtain MRI due to the limitation of the bore size of the magnet (typically 70 cm or less). Second, because of potential mechanical or electrical hazards, taking MRI from patients with metallic or electronic objects is difficult. However, imaging patients with wounds caused by metallic objects or together with interventional devices (for example, in the intensive care unit or the emergency room) is clinically desirable.

Ultra-low-field (ULF) MRI has been developed (for review, see [1]) as a potential solution to mitigate the above-mentioned challenges: ULF-MRI systems use magnetic field strengths in the range of micro- or milliteslas [2], making possible instrumentation at low cost, light weight, and open access. ULF-MRI systems have the advantages of metal compatibility [3] and high *T*_1_ contrast [4]. However, one major technical challenge of ULF MRI is its low signal-to-noise ratio (SNR). To address this issue, a separate stronger pre-polarization magnet has been suggested (in the range of tens of milliteslas) for magnetization generation while a weaker signal detection magnet (in the range of tens of microteslas) is used for magnetization precession. Additionally, highly sensitive superconducting quantum interference devices (SQUIDs) are typically used to detect the weak magnetic fields [2].

Since a SQUID array with up to tens or even hundreds of sensors can be used in an ULF-MRI system for signal detection, here we propose a data-processing procedure to reinforce data consistency among the collection of all spatially localized measurements from all SQUID sensors to suppress noise. In fact, this data consistency has been reported in high-field parallel MRI aiming at improving the spatiotemporal resolution at the cost of SNR [5]. However, in ULF MRI the SNR is the most important resource which cannot be compromised.

Different from aiming at achieving a higher spatiotemporal resolution, parallel MRI can exploit the redundancy among channels of a receiver RF coil array to suppress (motion) artefacts [6], [7], [8], [9], [10], [11] by pursuing the consistency in either *k*-space [6], [7], [10], image domain [9], or coil sensitivity maps [11]. Here, we attempt to exploit the redundancy among channels of a receiver coil array to improve the SNR of ULF MRI. Our method differs from previous approaches by using a universal kernel to enforce the data consistency among measurements across receiver coils iteratively and by adding *a priori* image sparsity information to further suppress the noise. Using simulations and experimental data, we demonstrate that our proposed method can effectively improve ULF-MRI image quality.

## Theory

### The correlation among the channels in parallel MRI detection

In the following, we use the word ‘coil’ to respectively indicate a pickup coil in the ULF-MRI system and a radio-frequency (RF) coil in high-field MRI. Accordingly, a coil array indicates the collection of pickup coils in ULF-MRI and RF coils in high-field MRI. Because the sensitivity map for each channel of a coil array is spatially smooth and distinct, the *k*-space data from each channel are locally and linearly correlated with data from other channels [5], [12], [13]. Specifically, using *d_i_*(**k** *_m_*) to denote the complex-valued *k*-space data at the *i* ^th^ channel and at *k*-space coordinate **k** *_m_*, we have
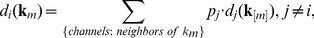
(1)where **k**_[*m*]_ denotes the *k*-space coordinates in the vicinity of **k** *_m_* (excluding **k** *_m_*). *p*_j_ are the fitting coefficients with the index *j* indicating different channels of the coil and neighbors of **k** *_m_*. Note that the vicinity here is related to the spatial smoothness of the coil sensitivity maps. The formulation above has been used extensively in high-field parallel MRI to increase the spatiotemporal resolution of MRI at the cost of SNR reduction (for a review, see [14]). For example, SMASH replaces the *k*-space data *d_i_*(**k** *_m_*) and *d_j_*(**k**_[*m*]_) with *B*_1_ sensitivity maps and desired spatial harmonic functions to derive the coefficients p*_j_*, which are later used to interpolate the missing *k*-space data [13]. GRAPPA uses the ‘auto-calibrated scan’ *k*-space data *d_i_*(**k**_m_) and *d_j_*(**k**_[m]_) to first estimate coefficients p*_j_*, which are then used to multiply the acquired *k*-space data in order to reconstruct the skipped *k*-space data [12]. Different from GRAPPA, which uses different *k*-space kernels to reconstruct data in different *k*-space lattice structures, SPIRiT is a method that synthesizes missing *k*-space data by using one single pattern to linearly correlate neighboring *k*-space data points from all receiver coils [5].

Eq. [1] has a matrix representation
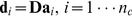
(2)where **d** *_i_* denotes a vector generated from the vertical concatenation of all *k*-space data points *d_i_*(**k** *_m_*) in channel *i*. **D** is a matrix, each row of which includes the *k*-space data points *d_j_*(**k**_[*m*]_) from all RF coils at *k*-space coordinates **k**_[*m*]_ in the neighborhood of **k** *_m_*. **a** *_i_* is a vector including the unknown coefficients. *n*_c_ is the number of channels in a coil array.

### Suppressing noise using data consistency

Instead of aiming at spatiotemporal resolution enhancement, we use Eq. [2] to enforce data consistency among channels of a coil array at all *k*-space locations. Without any noise contamination, Eq. [2] describes a linear dependency between a *k*-space data point and other *k*-space data points across all channels of a coil array. Specifically, given **d** *_i_* and **D**, **a** *_i_* can be estimated. Let 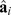 denote the estimate of **a** *_i_*. We expect that 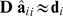.

To enforce such data consistency practically, we propose to iteratively 1) first estimate the coefficients 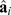, and 2) update all *k*-space data 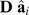. Specifically, at the *p* ^th^ iteration with data **D** *^p^* and **d** *_i_^p^*, we have 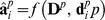, where 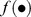 denotes the estimator. Subsequently, we update the data 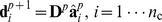 and 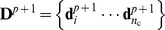. The procedure is repeated until convergence 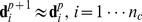.

The estimator 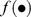 can be based on least-squares fitting for computational efficiency.
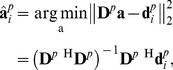
(3)where the superscript H denotes complex conjugate and transpose and 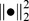 denotes the square of the l_2_-norm.

In addition, we can incorporate any prior information to estimate regularized coefficients 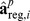 for channel *i* at an iteration step *p*.
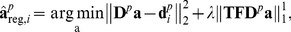
(4)where **F** denotes the Fourier transform, and **T** denotes taking the difference between a selected voxel and the average of its neighboring voxels. 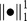 denotes taking the l_1_-norm. λ is a regularization parameter. The cost quantifying the ‘sparsity’ of the image in the transformed domain 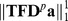 is closely related to the Total Variation [15], [16].

Eq. [4] was calculated in practice by the iterative re-weighted least-squares algorithm [17], which uses a computationally efficient weighted least-squares estimator with a diagonal weighting matrix changing over iteration to approximate the exact solution of Eq. [4]. Specifically, at the *j* ^th^ iteration, we have
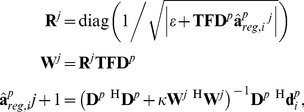
(5)where 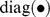 denotes generating a diagonal matrix from the vector argument. ε is a very small number to avoid divergence. The regularization parameter was determined by 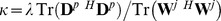, where 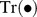 denotes the trace of a matrix. This is based on our previous studies in regularized parallel MRI reconstructions [18], [19], [20]. The λ used in this study ranged between 0.03 and 0.5.

## Methods

### Ethics Statement

All human images were acquired from subjects with written informed consent under the approval of the Institute Review Board of Aalto University.

**Figure 1:**
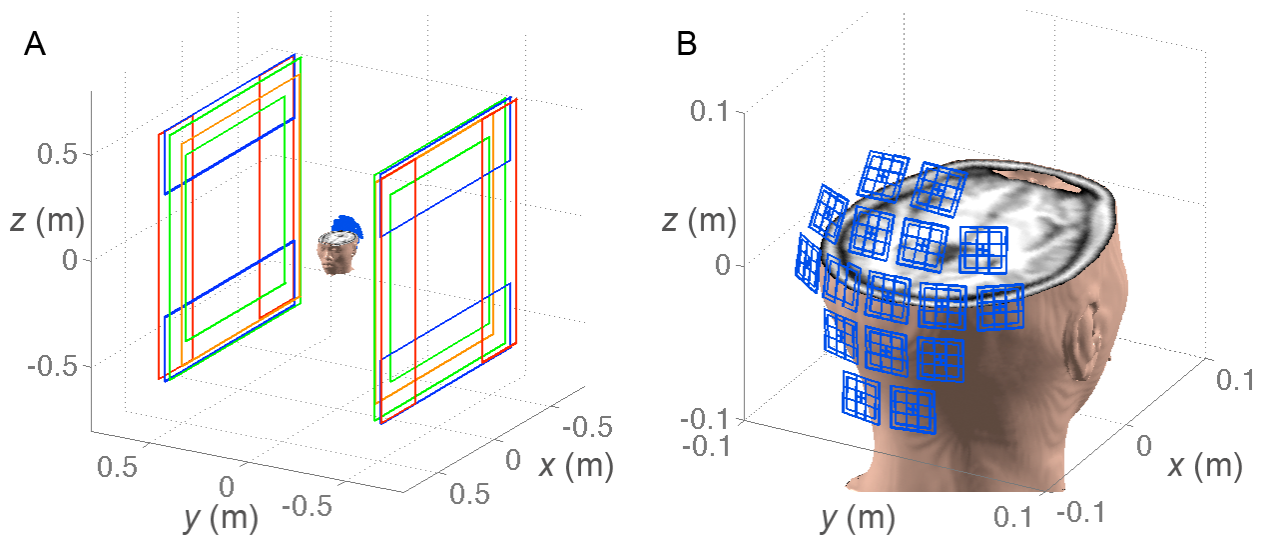
A: Our ULF-MRI system, which includes the *x*-, *y*-, and *z*-gradients (orange, red, and blue, respectively) and the magnet to generate the measurement field (green). The polarizing and excitation coils are not shown in the figure. B: The posterior view of the system shows 47 SQUID sensors covering the posterior parts of the head.

Our ULF-MRI system [21] has 47 SQUID sensors distributed over the occipital lobe in a helmet-shaped dewar (**Figure 1**). The field sensitivity of the sensors was 4 fT/√Hz for magnetometers and ∼4 fT/cm/√Hz for gradiometers. A constant *B*_0_ = 50 µT was applied for magnetization precession along the z direction in Figure 1. The configuration of this system was used for the subsequent simulations and empirical hand and brain imaging.

In the simulation, the ‘true image’ was based on a high-resolution *T*_1_-weighted head MRI acquired from a 3T MRI (Tim Trio, Siemens Medical Solutions, Erlangen, Germany). The data were acquired using a 32-channel head coil array with the MPRAGE pulse sequence (TR/TE/flip = 2530 ms/3.49 ms/7°, partition thickness = 1.0 mm, matrix = 256×256, 256 partitions, FOV = 256 mm×256 mm). One coronal-slice head image through the center of the brain was taken as the ‘true image’, which was then multiplied pixel-by-pixel by the individual coil-sensitivity map calculated by the Biot–Savart law after given the detection-array geometry (**Figure 1**) in order to simulate the noiseless detection **s** *_i_* in the *i* ^th^ channel of the coil array. To simulate acquisitions at different signal-to-noise ratios (SNRs), complex-valued Gaussian white noise **n** was scaled and then added to each channel of the coil array: SNR = √(**s** ^H^ **s**/**n** ^H^ **n**), where **s** is a vector consisting of **s** *_i_* across different channels. We simulated SNR = 0.5, 1, and 2 in this study.

**Figure 1** shows the schematic diagram of the ULF-MRI system. Experimental data were acquired using a 3D spin-echo sequence with TE = 80 ms to generate hand images of 6 mm×7.1 mm in-plane resolution (slice thickness 10 mm) using a maximal gradient strength of 85 µT/m. Before each *k*-space read-out measurement, the sample was polarized in a 22-mT field for 1 s. The total imaging time was 35 minutes. For brain images, we also used a 3D spin-echo sequence with TE = 122 ms, 4 mm×4 mm in-plane resolution (slice thickness 6 mm), and a maximal gradient strength of 130 µT/m. Before each *k*-space read-out measurement, the sample was polarized in a 22-mT field for 915 ms. The total imaging time was 90 minutes.

We also measured saline phantom images in the brain imaging experiment. This allowed us the estimates of coil sensitivity maps and consequently to use the non-accelerated SENSE MRI reconstruction algorithm [22], [23] with regularization [18], [20], [24] to optimally combine complex-valued coil images. Specifically, the regularization parameter λ was chosen as described in our previous study [21]. For images without sensitivity maps, we calculated the sum-of-squares images as the final reconstruction. To quantify the image quality, we calculate the peak signal-to-noise ratio (pSNR) of the image as the ratio between the largest pixel value and the background noise fluctuation, which was the square root of the mean of the image pixel values outside the imaging object. Since there is no golden standard in the empirical data, we also calculate the mean-square-error (MSE) of the reconstruction with respect to the reconstruction using all available averages. Data were reconstructed with Matlab (*Mathworks*, Natick, MA, USA) using in-house codes on a PC (two quad-core processors with 16 gigabytes of memory).

## Results

### Simulations

**Figure 2:**
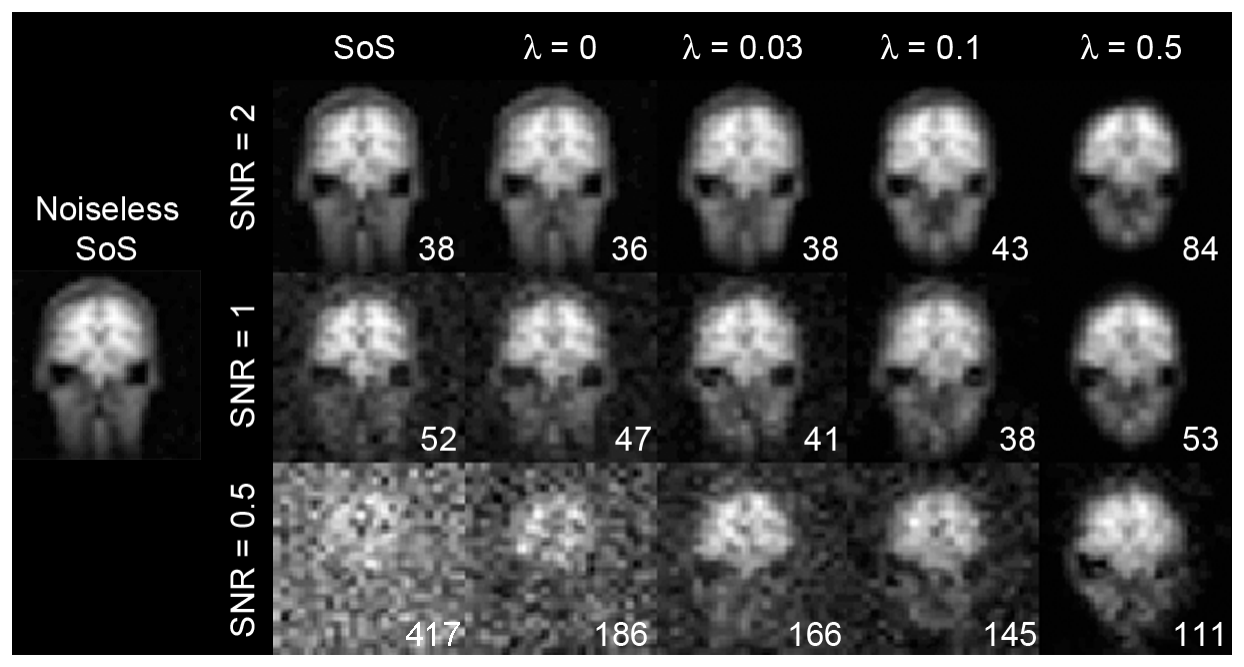
The simulated noiseless sum-of-squares (SoS) image from all 47 channels of the ULF-MRI system (left). At different SNRs compared to the direct SoS reconstruction, SNR can be improved by incorporating the data consistency constraint (λ = 0). Using the sparsity prior (λ = 0.03, 0.1, and 0.5), the residual error can be further reduced with low SNR acquisitions. The residual errors are reported at the lower-right corner of each reconstruction.

**Figure 2** shows the simulation results of reconstructing sum-of-squares (SoS) images. For comparison, a noiseless SoS reconstruction is also shown. Generally, these results show that the images become less distinguishable as SNR degrades. Using the data consistency constraint alone without any prior (λ = 0), we observed that the residual errors, quantified by the sum of the squares of the difference image between each reconstruction and the noiseless SoS image, decrease at all SNRs. The residual errors are also shown in the lower right corner of each reconstructed image. We also used the prior information of image sparsity to further suppress noise with three λ's (λ = 0.03, 0.1, and 0.5). The λ corresponding to the minimal residual error was found at different SNRs. At SNR = 2, the prior did not help reduce the residual error, because image features of lower intensity, such as the skull and muscles outside brain parenchyma, were also suppressed. At SNR = 1, data consistency constraint shows a smaller residual error. Using the sparsity prior, we found that λ = 0.03 gives the least residual error (approximating the best reconstruction at SNR = 2 with λ = 0) by showing some brain structures and low background noise. At SNR = 0.5, although the data consistency constraint and the prior of image sparsity can reduce the noise, the reconstruction is generally too noisy to discern the actual image features. However, surprisingly, with λ = 0.5, we can still delineate the brain parenchyma, which cannot be recognized in the original SoS and the SoS reconstruction using data consistency constraint alone. Taken together, at SNR>2 the data consistency constraint can suppress the noise. At SNR ∼1, the image can be better reconstructed using both the data consistency constraint and the prior information, assuming the image can be sparsely represented.

### Hand images

**Figure 3:**
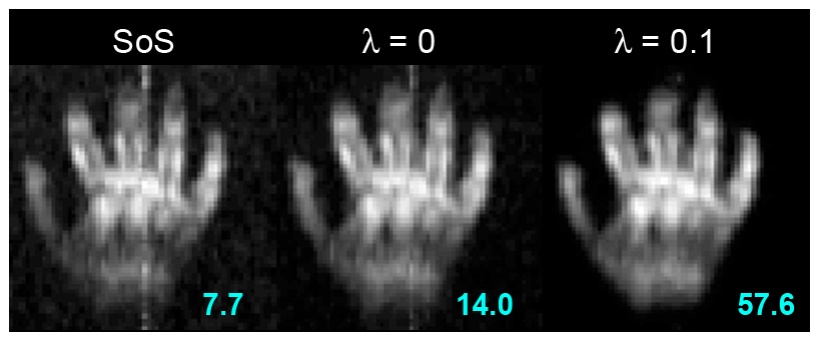
A hand sum-of-squares (SoS) image (left). The data consistency constraint (λ = 0) reduces significantly the noticeable vertical strip artifact (middle). Further, the sparsity prior (λ = 0.1) improves the reconstruction only marginally (right). The pSNR was indicated in each image.

**Figure 3** shows experimental images of the right hand of a subject. Without coil sensitivity measurement, we showed the sum-of-squares (SoS) image of five digits and the palm. Notably, there was a clear vertical strip artifact in the SoS image, potentially due to the SQUID noise at 3 kHz in our system. The background noise σ was 0.021. Using the data consistency constraint alone (λ = 0) reduced the vertical strip artifact and the background noise (σ = 0.012) significantly. Applying the data consistency constraint also increased the pSNR from 7.7 to 14.0. Further, the use of the sparsity prior (λ = 0.1) gave a similar reconstructed image as the reconstruction with λ = 0. The pSNR was further improved to 57.6 because of the strong suppression of the background noise.

### Brain images

**Figure 4:**
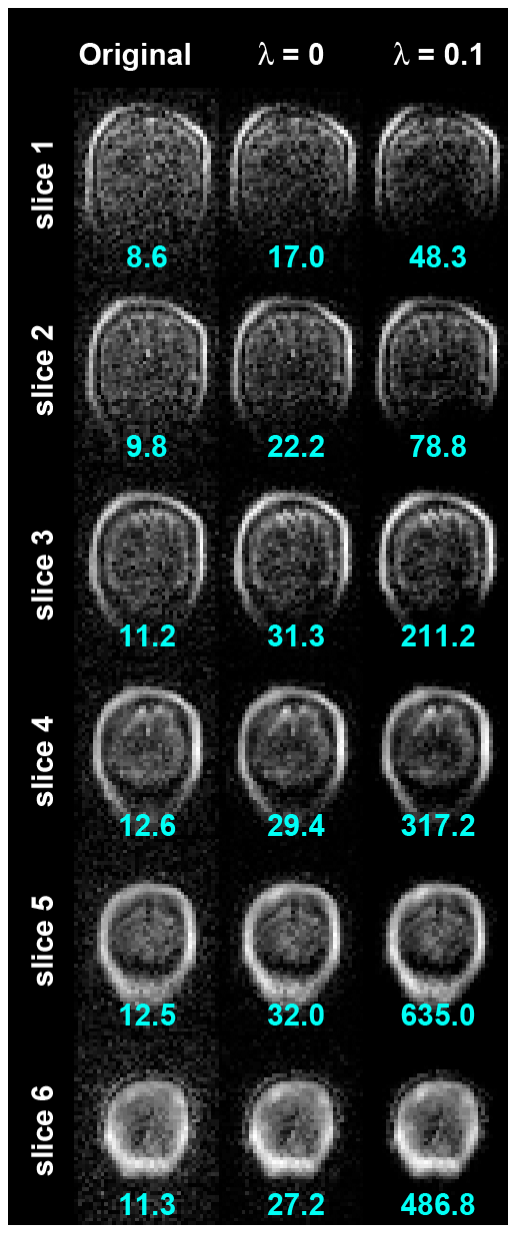
Brain images reconstructed by the regularized SENSE reconstructions with no acceleration (left column). The data consistency constraint (λ = 0) improves the image by showing a strong signal in the brain parenchyma (middle column). Further, the sparsity prior (λ = 0.1) suppresses the background noise significantly to better delineate the skull and the brain (right column). The pSNR was indicated in each image.

**Figure 5:**
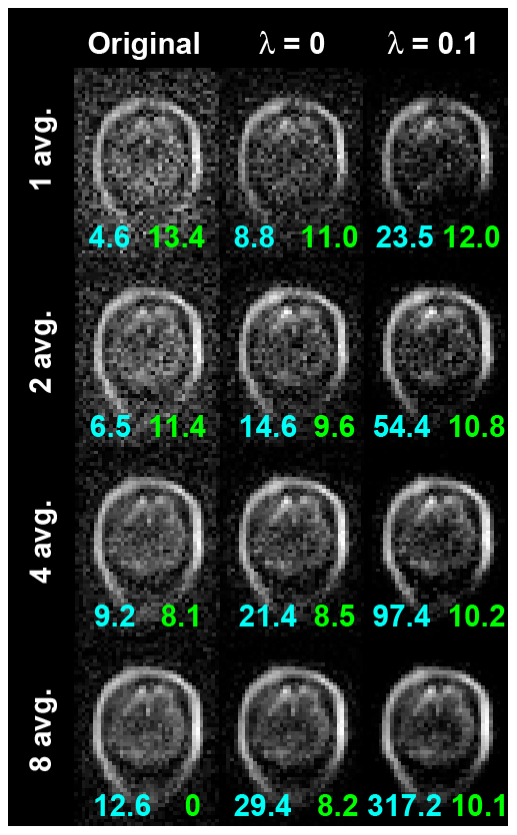
A brain image with different number of averages reconstructed by the regularized SENSE reconstruction with no acceleration. The pSNR (cyan) and MSE (green) were reported in each image.

Six coronal slices of brain images from our ULF-MRI system with 22-mT polarization, 130-µT/m maximum gradient, and 90-minute imaging time (eight averages) are shown in **Figure. 4**. The shapes of the skull and brain parenchyma were observed in the regularized SENSE reconstructions. We found that signals potentially from gray and white matter increased as the data consistency constraint was applied (λ = 0). The average pSNR across six images increased from 11 to 26. Furthermore, when the sparsity constraint was added, the average pSNR dramatically increased to 296. This was due to strong suppression of the background noise. However, applying the sparsity constraint also decreased the image intensity at the FOV center. **Figure 5** shows the regularized SENSE reconstruction of slice 4 using data with 1, 2, 4, and 8 averages. The pSNR increased in proportion to the number of averages for the original data. Using the same data, reconstructions that applied the data consistency constraint with λ = 0 had a 2.2-folds pSNR improvement. Specifically, the pSNR of the reconstruction with four averages gave similar pSNR to the reconstruction using unaveraged data with the data consistency constraint. This is similar to the 8-average data and 2-average data with the data consistency constraint. Using the sparsity constraint with λ = 0.01 further improved the pSNR by a factor of 12. However, one should be cautious that a higher pSNR should be further validated by golden standard images if possible. The MSE with respect to the regularized SENSE reconstruction with eight averages were also reported in **Figure 5**. Similar MSE results can be obtained by either the data consistency constraint or double the measurement time.

## Discussion

Our simulation and empirical results demonstrate that the use of the data consistency constraint in multiple-sensor ULF MRI can reduce the noise level and thus increase the (peak) SNR of the reconstructed images. Parallel imaging at high field using this data consistency constraint has been previously explored for imaging acceleration, where SNR is traded-off for the enhanced spatiotemporal resolution [5]. Without acceleration, the data consistency constraint has also been used to suppress motion artifacts in high-field MRI [6], [7], [8], [9], [10], [11]. While our method similarly keeps all the measurements in order to minimize the SNR loss, the algorithm is different from previous methods because 1) a universal kernel is used to enforce *k*-space data consistency, 2) an iterative procedure is used to enhance the data consistency progressively, and 3) prior information about image sparsity is included in the processing. Different from high-field MRI, where the dominant noise source is the imaging object, ultra-low-field MRI has dominant noise source from the instrument, including sensors and the dewar [1]. However, it should be noted that our algorithm is completely data-driven and does not depend on whether the noise comes dominantly from the instrument or the imaging object: any fluctuation deteriorating the *k*-space data consistency is suppressed by our method. One limitation of our method is that SNR cannot be improved when noise is correlated between channels, because the *k*-space data consistency is not disturbed in this special case. Without discarding any acquired data, the reconstructed image does not lose any SNR. By further adjusting the dependency among *k*-space measurements, we can even suppress the noise and thus obtain a higher SNR than in the original measurements.

It should be noted that since our algorithm only adjusts a given data set iteratively, the results will not be improved if the input is very noisy (Figure 2). The other limitation is that our method will not improve the image indefinitely over the iterations. Note that while our reconstructed images still contain noise, the algorithm does *not* introduce any distortion, because neither the *B*_0_ field nor the *k*-space sampling grid, two common factors introducing distortion in high-field MRI, was changed.

Our ULF-MRI system is based on a magnetoencephalography (MEG) system, which uses an array of SQUID sensors to detect the extracranial weak magnetic fields elicited by synchronous post-synaptic currents from mainly pyramidal cells [25]. Noise suppression is a critical procedure in both ULF-MRI and MEG data processing. Our method is different from the signal-space projection (SSP) [26] and signal-space separation (SSS) [27], [28] methods in MEG processing, both of which are spatial filtering methods to separate measurements into signal and noise components and to remove the latter. The data consistency constraint, however, is based on the *k*-space formulation, which is a unique property in MRI (MEG does not have similar spatial encoding). However, we expect that this method can be integrated with SSP and SSS to further suppress noise and thus to improve the quality of ULF MRI.

By adding the *a priori* information that an image can have a sparse representation, the image noise can be further suppressed. This is particularly advantageous for low-SNR images (**Figure 2**). The sparsity assumption was first incorporated to MRI as compressed sensing [15] and recently has been applied to MR angiography [15], [29], [30], [31], dynamic imaging [32], [33], [34], hyperpolarized MRI [35], [36], [37], chemical shift imaging [35], [38], [39], and relaxometry mapping [40], [41]. Our results suggest that such a sparsity assumption can be appropriately integrated with the data consistency constraint to further suppress the noise level in highly parallel MR signal detection. However, the over-reliance on the sparsity constraint (a larger λ parameter in our study) causes the loss of image features with a lower contrast (**Figure 2**), corroborating the side-effect reported in an earlier compressed sensing MRI study [15].

Accordingly, tuning of the regularization parameter can be critical to obtain optimal performance. Previously, in parallel MRI reconstruction with Tikhonov regularization, aiming at simultaneously minimizing the measurement error and deviating from the prior based on the l_2_-norm measure, we suggested the L-curve approach [24] and data-driven variance-partitioning approach [20] to optimize λ. However, the present study uses the l_1_ norm in evaluating the image sparsity (Eq. 4) and thus, for example, an L-curve cannot be calculated efficiently. Based on our heuristic simulations, we suggested that λ between 0.03 and 0.5 can generate satisfying results, yet the optimal λ depends on the SNR of the acquisitions. Other methods, such as L-curve [24], partitioning of the covariance matrix [20], and generalized cross validation [42], [43] may be used to estimate the appropriate regularization parameter.

Another issue related to incorporating the sparsity prior is the choice of a transformation to sparsify the image. Here we used the Total Variation based on a local Laplacian operator to sparsify the image. It is also possible to use the wavelet transform to achieve a sparse representation [15]. However, we found that the difference is marginal (not reported).

Our method is expected to be applicable to other MR measurements contaminated by noise. Thus, not only ULF MRI, but also other MRI applications suffering from noise contamination may benefit from this data consistency constraint. Yet the degree of improvement needs more systematic investigations.
